# The relationship between religiosity and meaningful work among Malaysian Muslim employees: The mediating role of existential labor

**DOI:** 10.1371/journal.pone.0279251

**Published:** 2022-12-15

**Authors:** Ja-Kyung Seo, Muzaffar Bin Mahudin, Young Woo Sohn

**Affiliations:** Department of Psychology, Yonsei University, Seoul, South Korea; KFUPM: King Fahd University of Petroleum & Minerals, SAUDI ARABIA

## Abstract

This study aimed to explore how religiosity affects the level of meaningful work among Malaysian Muslims, owing to Malaysia’s highly religious background. Although religiosity constitutes a major part of an individual’s value system, the influence of religiosity on the meaningfulness of work remains unclear. To address this gap, this study examined the indirect effects of the two types of religiosity—intrinsic religiosity (IR) and extrinsic religiosity (ER)—on meaningful work through existential labor, namely, surface acting and deep acting. Self-reported survey responses from 303 Malaysian Muslim employees were analyzed using structural equation modeling and bootstrapping analysis. The results showed that both surface acting and deep acting had significant mediation effects on the relationship between IR and meaningful work. By contrast, in the relationship between ER and meaningful work, surface acting’s mediation effect was not significant, whereas deep acting showed a positive mediation effect. Our findings suggest that even if employees share the same religion, meaningful work is shaped differently by the specific type of religiosity and those existential labor strategies that individuals develop. This study advances the understanding of the underlying psychological mechanisms of the impact of individual religious values in the workplace. Implications and limitations were discussed.

## Introduction

In most cultures, religion is a psychosocial driver that strongly influences personality, attitudes, and value systems and also determines and shapes social behavior [1]. Previous studies have indeed shown a positive relationship between religiosity and work variables, for example, in terms of organizational commitment, job satisfaction, organizational citizenship behavior, and employee performance [2–6]. However, limited studies have investigated the impact of religiosity on meaningful work, broadly defined as work that is personally significant and worthwhile [6, 7]. The relevance of religiosity in terms of meaningful work is worthy of further study as meaningful work is known to foster several critical work outcomes, namely, job satisfaction (e.g., [8]), work engagement (e.g., [9]), and employee retention (e.g., [10]). To address this research gap, we examined how an individual’s type and degree of *religiosity* affect the level of *meaningful work* among Malaysian Muslim employees. Two separate types of religiosity, *intrinsic religiosity (IR)* and *extrinsic religiosity (ER)*, were evaluated because previous studies have shown that they may have opposing influences on outcome variables [11].

We further attempted to identify the mechanisms that explain why and how religiosity is related to meaningful work by drawing on the conservation of resources (COR) theory [12] and the concept of “self-concept–job fit” [13]. Specifically, we propose that two types of *existential labor* [14], namely, *surface existential acting* and *deep existential acting*, mediate the link between religiosity and meaningful work, but in opposite directions [11].

Malaysia was selected as the research object because it is a highly religious country—approximately 60% of the population is Muslim, and Islamic religiosity plays a crucial role as a spiritual and behavioral guide in everyday life [15]. Islamic beliefs and practices are also embraced and manifested in work contexts [16]. For example, Muslim employees are required to practice *Dhuhr* (afternoon prayer) and *Asr* (evening prayer) at work and fast during Ramadhan. Therefore, Malaysia offers a suitable setting to explore whether and how individual religiosity spills over positively into the work domain.

Accordingly, this study had three aims: to (1) increase the knowledge of religiosity’s impact on meaningful work; (2) deepen the understanding of how and why religiosity relates to meaningful work by examining the mediating roles of existential labor; and (3) reveal how the types of religiosity and existential labor distinctively affect meaningful work. The results of our study can provide insights into the role of religiosity as a source of meaningfulness in the workplace.

### Theory and hypotheses development

#### Conflicting roles of intrinsic religiosity and extrinsic religiosity

Religiosity is defined as the strength of an individual’s connection to or conviction for their religion [17], and it has been shown to stimulate intrinsic strengths, such as trustworthiness, goal-setting behavior, subjective well-being, and self-control in employees [18, 19]. However, research has shown conflicting results regarding relationships between religiosity and certain psychological outcome variables [11, 20]. This inconsistency may be because the two types of religiosity, IR and ER, have been shown to have opposing influences on mental health [11, 21]. Intrinsic and extrinsic religious orientation represent different aspects of religiosity, each serving as favorable and unfavorable antecedents of the subsequent psychological factors [22]. By definition, individuals with high IR view religion as their primary source of motivation and the ultimate end goal in life. The personal goals and needs of those with high IR are often centered on what their religion dictates [11]. Intrinsic devotion to religion is particularly imperative in Islamic literature because it highlights the role of Islamic teachings as essential resources to manage life’s difficulties [23]. Alternatively, ER refers to using religion for instrumental purposes, for example, to obtain self-centered goals such as comfort, security, protection, personal profit, or social connections [24].

As intrinsically motivated individuals tend to be more sincere in their religious devotion [25] than extrinsically motivated individuals and fill their lives with intrinsic motivation and meaning [26], IR is negatively associated with anxiety and depression, whereas ER is positively associated with them [27]. Furthermore, another study showed that psychological well-being was positively related to IR, but negatively related to ER among undergraduates in Spain [28]. Likewise, according to a recent research, IR rather than ER functioned as the primary personal resource for coping with adversity during the COVID-19 pandemic [29].

#### Existential labor: Surface acting and deep acting as mediators

Studies have suggested that simply holding religious beliefs is insufficient to predict an individual’s social and work outcomes; a crucial factor is integrating their personal religious beliefs into their social context [6, 30]. Therefore, exploring the detailed process of integration, surface acting and deep acting in this case, between individual religiosity and the work context is necessary. These are the two subcategories of emotional labor, usually defined as the emotion regulation strategy adopted by an individual to conform to organizationally set display rules to achieve role expectations [31]. Employees either fake their emotional expressions (i.e., surface acting) or alter their inner feelings to match the required ones (i.e., deep acting) to manage their emotions [32].

Considering that our study aims to uncover the influence of individual religious beliefs on the meaningfulness of work, we focused on “self-regulation of meaningfulness exhibition” rather than “emotion regulation” when evaluating surface and deep acting [14]. In other words, emotional labor was replaced with *existential labor* to precisely convey our purpose. An employee’s emotional labor refers to the stress of managing their emotions when their organization requires them to exhibit certain behaviors and values to clients or co-workers [33]. Meanwhile, existential labor is the regulatory effort to fill the gap between an individual’s sense of meaningfulness and that displayed by their employer [14]. Employees derive their decisions and actions from their values, but these values may be at odds with the organization’s value norms that guide employees’ behavior [34]. To address this gap, employees either act as if they endorse the organizational values while suppressing their true values (i.e., surface acting) or proactively align with and make sense of the required meaningfulness (i.e., deep acting) [14].

Faking or suppressing true emotions consumes significant psychological resources; hence, surface acting entails more resource depletion than deep acting [32, 35]. Deep acting minimizes psychological costs by reducing the discrepancy between felt and expressed emotion. However, the reverse may be true for existential labor because deep acting requires a conscious investigation into and modification of an individual’s deep sense of meaningfulness. This requires significant investments of personal resources far more than emotion regulation [14].

Drawing on the COR theory [12], we propose that the depth of employees’ religiosity determines their propensity to engage in surface or deep existential acting. The COR theory posits that insufficient resources lead to defensive attempts to avoid the immediate loss of resources, whereas sufficient resources encourage active resource investment for further accumulation. Although deep acting may consume more resources than surface acting in the short term, individuals with high levels of personal resources are more likely to choose deep acting rather than surface acting because, of the two types, deep acting is a more effective strategy with positive consequences (e.g., enhanced self-efficacy and less burnout), once a congruent state is achieved [35].

We argue that IR can contribute to the personal resources and perseverance required for deep acting because high IR has been associated with successful coping with adverse situations in the workplace [18, 29]. For example, Islamic teachings provide interpretations of difficulties in life that individuals can use as resources to manage their difficulties [23]. Hence, we propose that Muslim employees with a high degree of IR, who are thus equipped with more positive personal resources than those with low IR, are more likely to engage in deep acting. By contrast, employees with a high ER would choose surface acting because their religiosity originates less from intrinsic motivation and would therefore have fewer resources to implement a deliberate cognitive strategy, such as deep existential acting [14]. Individuals with a high IR, and thus with more personal resources, would be less attracted to surface acting due to the potential loss of future resources. In contrast, individuals with a high ER would probably prefer surface acting, which requires less immediate resource input than deep acting; hence, we proposed Hypothesis 1:

Hypothesis 1a. IR is positively related to deep acting but negatively related to surface acting.
Hypothesis 1b. ER is negatively related to deep acting but positively related to surface acting.

#### Existential labor and meaningful work

The concept of self-concept–job fit [13] argues that when an individual’s work experiences match their self-concept, they perceive their work as meaningful. This congruence between self-concept and work is an antecedent of meaningful work because individuals seek an environment that helps maintain a consistent self-concept [13, 36]. Notably, the self-concept–job fit was a stronger predictor of meaningful work than other traditional types of person–job fit, which are perceptions of demands–abilities, supply–value, and person–organization fit [13]. Since the self-concept consists of beliefs and values related to personal characteristics and roles [37], the meaningfulness of work should increase when it aligns with an employee’s religious values. Social psychological research also claims that individuals strive to engage in behaviors that help them maintain a sense of consistency [38]. Specifically, employees align individual identities and work situations through identity work, such as emphasizing their preferred work identities that concur with their present work experiences [36]. In our view, deep existential acting is an identity work strategy that employees can adopt to achieve an alignment between their self-concept and work and, thus, meaningful work [14]. Through deep acting, employees can alter their perceptions and attitude toward work situations, increasing their level of perceived self-concept–job fit. For this reason, we assumed that Malaysian Muslims engaging in deep-acting processes would be more likely to find their work meaningful than those who do not. However, surface acting would hinder the forming of meaningful work because faking consumes a considerable amount of emotional energy, engendering negative emotions and exhaustion [14, 39]. In line with our assumption, previous studies have also found that surface-acting strategies have negative relationships with job satisfaction but positive relationships with stress and exhaustion, while deep-acting strategies show a reverse pattern [40]. Therefore, we hypothesize as follows:

Hypothesis 2a. Deep acting is positively related to meaningful work.
Hypothesis 2b. Surface acting is negatively related to meaningful work.

#### Religiosity and meaningful work: A mediation model

Exploring the joint impact of personal and environmental sources on the meaningfulness of work and understanding how they relate to each other in a single model are crucial [10, 41]. To address this research question, we combined the arguments on the relationships between religiosity, existential labor, and the meaningfulness of work and hypothesized that IR indirectly improves the meaningfulness of work by increasing deep acting and decreasing surface acting. Conversely, we propose that ER decreases the meaningfulness of work indirectly by decreasing deep acting and increasing surface acting. Thus, Hypothesis 3 is as follows:

Hypothesis 3a. The positive relationship between IR and meaningful work is mediated by deep acting and surface acting.
Hypothesis 3b. The negative relationship between ER and meaningful work is mediated by deep acting and surface acting.

A recent study demonstrated that religiosity is positively and significantly associated with meaningful work [6]. In this study, we attempt to delve deeper and clarify the mechanism by which religiosity influences meaningful work by investigating the disparate effects of intrinsic and extrinsic forms of religiosity on the meaningfulness of work among Malaysian Muslim employees. Additionally, we explore the mediating role of individual–work value alignment strategies, specifically surface and deep acting, in the relationship, and thereby, expand the understanding of the influence of religiosity on the meaningfulness of work and the role of existential labor.

## Method

### Participants and procedure

A cross-sectional study was conducted in May 2021. A link to the online survey was shared on several online platforms, namely, Twitter and LinkedIn, inviting Malaysian employees to participate in this study. The participants voluntarily provided their written informed consent online before beginning the survey; furthermore, no compensation was provided for completion. At the beginning of the survey, the questionnaire presented two screening questions asking participants for their religion and employment status to screen out those who were not Muslim or were not employed full-time. A total of 346 full-time Muslim employees completed the survey and we excluded those who failed to correctly answer a bogus question. Thus, the final sample of 303 participants was used for the following statistical analyses. This study received ethical approval from the Institutional Review Board (IRB) at Yonsei University (IRB# 7001988-202201-HR-1115-04).

The participants had an average age of 28.39 years (*SD* = 4.50) and average job tenure of 3.30 years (*SD* = 3.60). Among the participants, 21.1% were male (*N* = 64), 87.1% (*N* = 264) had completed a bachelor’s or master’s degree, approximately two–thirds (78.2%, *N* = 237) were single, and the majority identified themselves as supporting staff or associates (89.1%, *N* = 270).

### Measures

The *IR* and *ER* were measured using 14 items from the Intrinsic/Extrinsic Revised Scales (I/E-R Scales) [24]. The IR and ER were assessed using eight (e.g., “I have often had a strong sense of God’s presence”) and six items (e.g., “I pray mainly to gain relief and protection”), respectively. Both subscales were rated on a 5-point Likert scale ranging from 1 (“strongly disagree”) to 5 (“strongly agree”). The reliability and the corrected item–total correlation of the items for each dimension were computed, resulting in the removal of the sixth item (“Although I am religious, I don’t let it affect my daily life”) of the IR scale due to its low corrected item–total correlation (.06) [42]. The sixth item of ER (“I go to mosque mainly because I enjoy seeing individuals I know there”) was also removed, because the confirmatory factor analysis (CFA) later demonstrated that its standardized factor loading was very low (< .20). Accordingly, the Cronbach’s alphas for the final items were .70 for IR and .74 for ER.

*Existential labor* was measured using the Emotional Labor Scale (ELS) [43]. For this study, we adopted and revised two of the six ELS subscales, surface acting and deep acting. Three items assessed surface acting (e.g., “I resist expressing my true values and beliefs”), and the other three items assessed deep acting (e.g., “I make an effort to actually feel the values and beliefs that I need to display to others”). Both subscales were rated on a 5-point Likert scale ranging from 1 (“never”) to 5 (“always”). All six items were subjected to exploratory factor analysis using a varimax rotation for factor structure verification. As expected, the results revealed the presence of two factors with eigenvalues greater than 1. The two-factor structure explained 57.8% of the total variance, and all items had primary loadings over .5 without a cross-loading above .3. The Cronbach’s alphas were .72 and .84 for surface acting and deep acting, respectively.

*Meaningful work* was measured using the Work and Meaning Inventory [44], comprising three subscales: positive meaning (4 items), meaning-making through work (3 items), and greater good motivations (3 items). Example items are, “I have found a meaningful career,” “My work helps me better understand myself,” and “The work I do serves a greater purpose.” All subscales were rated on a 5-point Likert scale from 1 (“absolutely untrue”) to 5 (“absolutely true”); Cronbach’s alpha was .91.

We performed Harman’s single-factor test to manage the limitation of the common method variance, since the data were collected through self-reports [45]. We found that the total variance extracted by one factor was 19.72%, less than the recommended threshold of 50%, indicating the absence of common factor bias in the data.

### Statistical analysis

Before model testing, SPSS v25.0 was used to compute descriptive statistics, bivariate correlations, and reliability estimates. The measurement model and the hypothesized mediation model were examined by structural equation modeling using Mplus version 8.6. When testing the structural model, sociodemographic variables, namely, gender (0 = male, 1 = female), job tenure, job income (four dummy coded variables), marital status (0 = single, 1 = married), and education (four dummy coded variables) were controlled. The model was estimated with the robust maximum likelihood method and the results were examined with the indices of model fit, namely, the standardized root mean square residual (SRMR), comparative fit index (CFI), Tucker–Lewis index (TLI), and root mean square error of approximation (RMSEA). A model is considered good if the SRMR is less than .08, the CFI and TLI are .90 or greater, and the RMSEA is lower than .08 [46]. Finally, we conducted bootstrapping with 5,000 bootstrap samples and 95% bias-corrected CIs using Mplus to test the significance of the hypothesized direct and indirect paths [47]. Because the normality assumption of the indirect effect is not fulfilled, using the bootstrap method is more strongly recommended than the Sobel test and significance testing [48].

## Results

### Descriptive statistics

Correlations were computed to examine the extent to which the study variables were associated. As shown in Table 1, IR and ER had a small positive correlation (*r* = .28, *p* < .001). Deep acting was significantly and positively correlated with IR (*r* = .31, *p* < .001) and ER (*r* = .26, *p* < .001), and surface acting had a significant negative association with IR (*r* = −.30, *p* < .001). Meaningful work showed a significantly negative association with surface acting (*r* = −.16, *p* < .01), but a significantly positive relationship with deep acting (*r* = .24, *p* < .001). No significant associations were found between meaningful work and both types of religiosity, IR (*r* = .09, *p* > .05) and ER (*r* = .07, *p* > .05).

**Table 1:** Means, standard deviations, correlations, and Cronbach’s alphas for the main variables.

Variables	1	2	3	4	5	6
1. Intrinsic religiosity	(.70)					
2. Extrinsic religiosity	.28**	(.74)				
3. Deep acting	.31**	.26**	(.84)			
4. Surface acting	−.30**	.07	−.13*	(.72)		
5. Meaningful work	.09	.07	−.16**	.24**	(.91)	
6. Job identification	.03	.20**	−.01	.15*	.56**	(.84)
*M*	3.98	3.25	2.43	3.86	3.83	3.98
*SD*	0.63	0.71	0.90	0.77	0.71	0.89

Note. *N* = 303; Numbers in parentheses are Cronbach’s alpha reliability coefficients.

*p < .05.

**p < .01.

### Hypotheses testing

#### Measurement model

The measurement model was first tested by CFA to evaluate how well the observed items represented the latent variables and distinction between the key constructs (i.e., IR and ER, surface and deep acting, and meaningful work). For factors with more than four observed items, parcels were created because item parceling results in more precise parameter estimates with less sampling error and bias than using item-level data [49]. Using the factorial algorithm technique [49], 10 meaningful work items were transformed into three parcels, and seven IR and five ER items were each transformed into two parcels. The measurement model showed a good fit to the data: χ^2^ (55) = 77.63, *p* < .05; CFI = .987; TLI = .981; RMSEA = .037; SRMR = .039. All parcels and items significantly loaded onto their respective factors (range λ = .54–.93; *p* < .001), demonstrating convergent validity.

#### Structural model

**Fig 1:**
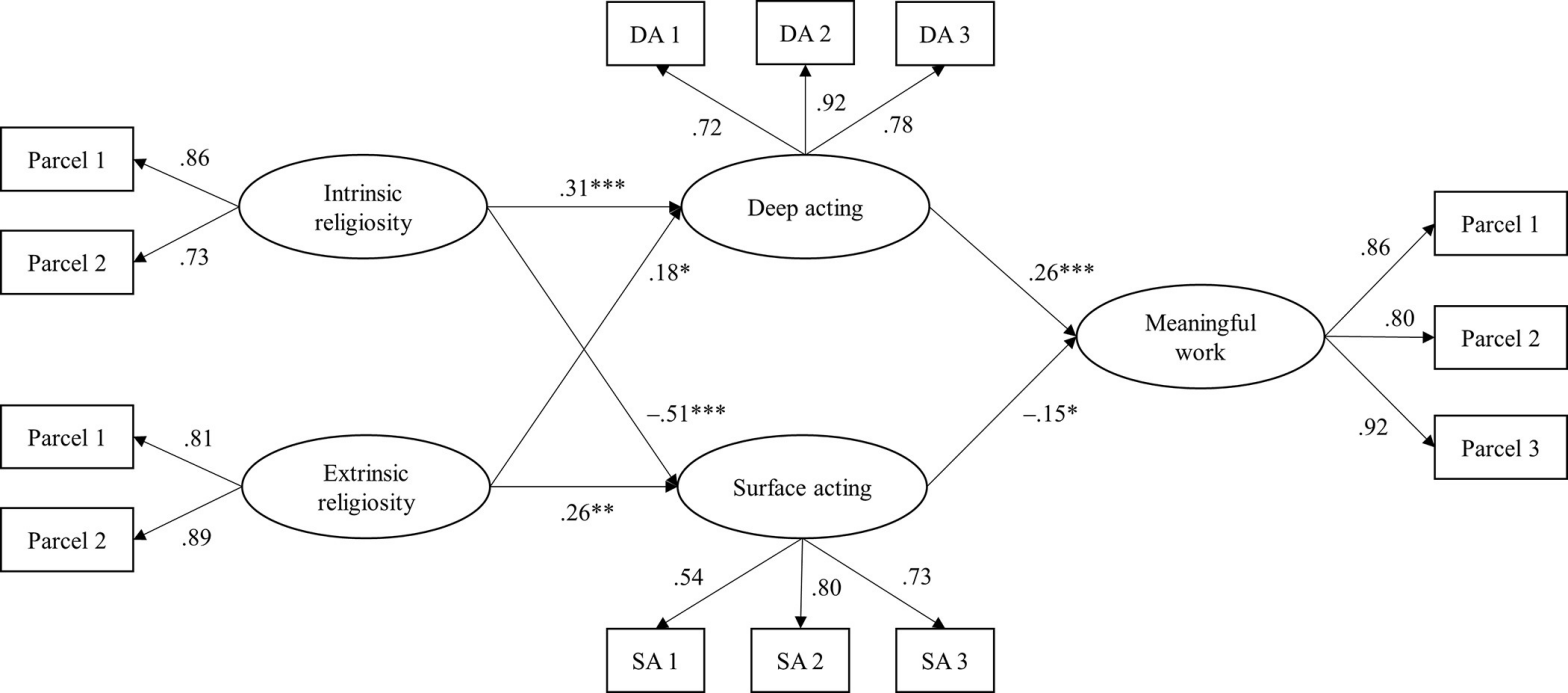
Structural equation model of relationships between IR, ER, deep acting, surface acting, and meaningful work while controlling for demographic variables. *Note*. Standardized path estimates are reported. All factor loadings from the latent to observed variables are significant at the p < .001 level. *p < .05, **p < .01, ***p < .001.

To test the hypothesized indirect effects, we estimated structural models in which the paths from IR and ER to meaningful work were fully mediated by surface and deep acting. First, the results of the full mediation model demonstrated a good fit to the data (*Χ^2^* (118) = 191.58, *p* < .001; CFI = .958, TLI = .949, RMSEA = .045, SRMR = .065). Next, we found the best-fitting model by adding the paths from IR and ER to meaningful work to specify a partial mediation model. This model also provided a good fit (*Χ^2^* (116) = 190.07, *p* < .001; CFI = .957, TLI = .948, RMSEA = .046, SRMR = .061). To compare these two nested models, we performed a chi-square difference test. The result confirmed that the model fit of partial mediation was not significantly better than that of the original full mediation model (∆ *Χ^2^* = 1.51, ∆ *df* = 2, *p* > .05). The standardized path coefficients of the full mediation model are illustrated in Fig 1. IR was positively associated with deep acting (*β* = .31, *p* < .001) but negatively associated with surface acting (*β* = −.51, *p* < .001). Contrary to Hypothesis 1b (i.e., ER was positively related to surface acting but negatively related to deep acting), ER was positively associated with surface acting (*β* = .26, *p* < .01) and deep acting (*β* = .18, *p* < .05). As predicted in Hypotheses 2a and 2b, surface acting was negatively related to meaningful work (*β* = −.15, *p* < .05), but deep acting was positively related to meaningful work (*β* = .26, *p* < .001). Although not shown in Fig 1, among the control variables, job income (*β* = .15, *p* < .05) and marital status (*β* = .12, *p* < .05) affected meaningful work significantly.

To examine the significance of the mediation effects of deep acting and surface acting between the two types of religiosity and meaningful work, as Hypotheses 3a and 3b predicted, bootstrapping was conducted with 5,000 samples to create 95% bias-corrected confidence intervals (CIs) [47]. If zero is not included within the lower and upper CIs, the effect is considered significant at the *p* < .05 level [50]. In Table 2, our bootstrapping analyses revealed a significant positive indirect effect of IR on meaningful work via surface acting (bootstrap estimate = .08, 95% CI [.002, .148]) and deep acting (bootstrap estimate = .08, 95% CI [.032, .139]). The indirect effect of ER on meaningful work via deep acting was in a positive direction (bootstrap estimate = .05, 95% CI [.007, .101]) because contrary to Hypothesis 1b, ER was positively associated with deep acting (*β* = .18, *p* < .05). Conversely, bootstrapping analyses showed that the indirect path from ER to meaningful work via surface acting was negative, as predicted, but nonsignificant because 95% bias-corrected CIs included zero (bootstrap estimate = −.04, 95% CI [−.088, .001]).

**Table 2:** Indirect effects of IR and ER on meaningful work through surface acting and deep acting.

Indirect Effects	β	CI [LLCI, ULCI]
IR → Deep acting → Meaningful work	.08**	[.032, .139]
IR → Surface acting → Meaningful work	.08*	[.002, .148]
ER → Deep acting → Meaningful work	.05*	[.007, .101]
ER → Surface acting → Meaningful work	−.04	[−.088, .001]

*Note*. Standardized coefficients and 95% confidence intervals are displayed.

IR, intrinsic religiosity; ER, extrinsic religiosity; CI, confidence interval; LLCI, lower limit confidence interval; ULCI, upper limit confidence interval.

*p < .05

**p < .01.

## Discussion

The main purpose of this study was to explore the link between religiosity and meaningful work and to investigate the mediating roles of existential labor (surface acting and deep acting) among Malaysian Muslim employees. Despite its prevalence and importance in value systems, limited studies have empirically examined the association between religiosity and meaningful work [7] or meaning-making [6]. Thus, this study integrated COR theory [12] and the concept of self-concept–job fit [13] to examine whether different religiosity types (i.e., IR and ER) yielded dissimilar impacts on employees’ following existential labor strategies and meaningful work.

### Theoretical contributions

Our study aimed to contribute to the literature by examining whether a personal spiritual life represented by religiosity spills over positively into the meaning of daily work [51]. According to our literature review, this study is the first to reveal that the effect of religiosity on meaningful work may differ through an individual’s existential labor strategy. We demonstrated the disparate mediating roles of deep and surface acting, adding evidence to the proposed ambivalent relationship between religiosity and meaningful work. The opposite effects of IR and ER demonstrated in this study corroborated findings by previous studies on religiosity (e.g., [11]). Thus, this study advances the understanding of the distinct roles of IR and ER in the work domain by verifying the mediation hypotheses. Overall, these findings expand the knowledge of how Muslim employees with religiosity can gain meaning in their workplace.

The results provided valid support for our proposed model except for a few unexpected findings. Specifically, Hypothesis 1a (IR is positively related to deep acting, but negatively related to surface acting) was fully supported; however, Hypothesis 2b (ER is negatively related to deep acting, but positively related to surface acting) was only partially supported. In line with the literature indicating that IR is associated with positive psychological outcomes [21, 24, 27], IR decreased surface acting but increased deep acting as expected. This finding supports and extends the COR theory [12] by revealing that an individual’s genuine belief in religion can act as one of many personal resources and can facilitate further acts of self-regulation in the work environment. Based on the COR theory, our finding implies that those with high religious beliefs have sufficient internal resources required for their long-term benefit even when an immediate loss of resources is expected. In our study, Muslim employees with genuine belief in their religion not only abided by their Islamic teachings in their workplace but also aligned their religious values with the organizational value norms. Furthermore, these findings indicate that deep existential acting requires far more personal resources than surface existential acting as it involves a complicated cognitive adjustment process. The opposite is true for deep and surface emotional acting because the latter drains more psychological resources to cope with the discrepancy between felt and expressed emotion [32]. Hence, our study is one of the first to demonstrate the difference between emotional and existential labor.

Notably, ER significantly increased both deep and surface acting, contrary to our prediction that ER would negatively affect deep acting. This finding is interesting as it suggests that ER can simultaneously have both positive and negative effects on subsequent employee behavior. In other words, it alludes to the possibility that employees with high ER might employ both existential labor strategies when adapting to the organizational values. A probable reason might be that ER can replenish the required personal resources for conducting deep acting when religious activities fulfill the coveted self-centered goals (e.g., social needs and emotional comfort). According to the COR theory, any opportunities that replenish psychological resource pools can offset the difficulties of challenging conditions [12]. Indeed, the frequency of prayer and involvement in religious activities such as attending religious gatherings and going to the mosque were positively related to mental health among Muslim university students [23]. Otherwise, it may be the case that employees with high levels of ER perceive the discrepancy between religious and organizational values as being less significant than those with high levels of IR.

Our findings also supported Hypotheses 2a and 2b (deep acting would increase meaningful work, but surface acting would decrease meaningful work). The results demonstrated that the deep acting strategy, which resembles deliberate identity work [36] or meaning-making [52] efforts such as a positive reappraisal, made a positive contribution to perceiving work as worthwhile. However, surface acting degraded the level of meaningful work. Hence, as the concept of self-concept–job fit indicates, merely holding religious beliefs is insufficient to predict positive outcomes in managing organizational social contexts, but improving the self-concept–job fit might be the decisive factor [6, 14].

Finally, according to the mediation analyses, deep and surface acting differentially mediated the relationship between religiosity and meaningful work. Specifically, Muslim employees with a high IR tended to engage in deep acting more than surface acting. Consequently, they had higher levels of meaningful work than those with low levels of IR. Notably, the indirect effect of ER on meaningful work was positively significant when the mediation path incorporated deep acting as the mediator; however, it became negatively significant when surface acting was the mediator, partially supporting Hypothesis 3. In other words, depending on the levels of the two existential labor strategies adopted, Muslim employees with a higher level of ER reported either a higher or lower level of meaningful work than those with a lower level of ER. Hence, employees with high ER can also increase meaningful work but only when they engage in deep acting. This result is notable because it partially supports the extant literature and explains a neglected link through which ER can positively affect meaningful work.

One study revealed that religiosity was positively associated with OCB and work engagement [6], and this study adds to the literature by demonstrating that when proactively integrated and managed in work settings, individual religious beliefs generated meaning for work. Specifically, this study was one of the first to provide significant empirical evidence regarding the role of existential labor, which has emerged through theoretical advocacy [14].

### Practical implications

Encouragement of meaningful work is an important task for management because research has found that meaningful work alleviates negative outcomes such as alienation, cynicism, and turnover intention [53, 54] and has a positive association with employee engagement [8]. Hence, as this study offers concrete evidence on how religiosity can serve as a personal resource that fosters the formation of meaningful work, organizations should aim to ensure that work is meaningful. First, organizations should account for individual differences in religiosity and focus on recruiting individuals with high religiosity. Second, our findings imply that active engagement in deep existential acting, not merely religiosity, is critical to the development of meaningful work. Therefore, organizations should offer opportunities and resources necessary for religious employees to adopt deep acting. For instance, organizational efforts to promote a harmonious religious culture that espouses various religious backgrounds may enhance the fit between the self-concept and the workplace environment, alleviating the difficulties of a deep-acting strategy [14]. Providing work opportunities that integrate employees with their religious values may also increase the likelihood of their work and personal identity aligning, ultimately helping individuals to find their work meaningful [36].

### Limitations and suggestions for further research

This study has several limitations. First, the sample mainly consisted of young female Malaysian Muslim employees; therefore, it might not be generalizable. Thus, further research that uses demographic groups different from those in this study and geographic regions beyond Malaysia would be beneficial. Particularly, determining if the relationships between religiosity, existential labor, and meaningful work observed in this study could be replicated in samples from other religious backgrounds, such as Christianity and Buddhism, is necessary. Second, as this study was cross-sectional, causality among the study variables should be interpreted cautiously. Longitudinal studies are necessary to determine causality among the variables and further investigate how religiosity develops into subsequent job behaviors and attitudes in the longer term. Third, all our data were self-reported; thus, further research should use multiple measurement methods, such as peer reports, to avoid response bias. Finally, this study utilized and revised the preexisting ELS to measure existential labor. Although both exploratory factor analysis and CFA have indicated a good fit in this study, further research should examine the psychometric properties to determine whether this scale fully and accurately covers the construct of existential labor.

## Supporting information

S1 File Data religiosity and meaningful work. (XLSX)
